# Neuroprotective Potentials of Berberine in Rotenone-Induced Parkinson’s Disease-like Motor Symptoms in Rats

**DOI:** 10.3390/brainsci14060596

**Published:** 2024-06-13

**Authors:** Hsiang-Chien Tseng, Mao-Hsien Wang, Chih-Hsiang Fang, Yi-Wen Lin, Hung-Sheng Soung

**Affiliations:** 1 Department of Anesthesiology, Shin Kong Wu Ho-Su Memorial Hospital, Taipei 11101, Taiwan; 2 School of Medicine, Fu Jen Catholic University, New Taipei City 24205, Taiwan; 3 Department of Anesthesia, En Chu Kon Hospital, Sanshia District, New Taipei City 23702, Taiwan; 4 Department of Orthopedics, College of Medicine, Taipei Medical University, Taipei 110, Taiwan; 5 Institute of Biomedical Engineering, National Taiwan University, Taipei 10051, Taiwan; 6 Department of Psychiatry, Yuan-Shan Branch of Taipei Veteran General Hospital, No. 386, Rongguang Rd., Neicheng, Yuanshan Township, Yilan 26604, Taiwan; 7 Department of Biomedical Engineering, National Defense Medical Center, Taipei 11490, Taiwan

**Keywords:** berberine, rotenone, trigonelline, motor impairment, striatum

## Abstract

Rotenone (RTN) induces neurotoxicity and motor dysfunction in rats, mirroring the pathophysiological traits of Parkinson’s disease (PD), including striatal oxidative stress, mitochondrial dysfunction, and changes in neural structure. This makes RTN a valuable model for PD research. Berberine (BBR), an isoquinoline alkaloid recognized for its antioxidative, anti-inflammatory, and neuroprotective properties, was evaluated for its ability to counteract RTN-induced impairments. Rats received subcutaneous RTN at 0.5 mg/kg for 21 days, resulting in weight loss and significant motor deficits assessed through open-field, bar catalepsy, beam-crossing, rotarod, and grip strength tests. BBR, administered orally at 30 or 100 mg/kg doses, one hour prior to RTN exposure for the same duration, effectively mitigated many of the RTN-induced motor impairments. Furthermore, BBR treatment reduced RTN-induced nitric oxide (NO) and lipid peroxidation (LPO) levels, bolstered antioxidative capacity, enhanced mitochondrial enzyme activities (e.g., succinate dehydrogenase (SDH), ATPase, and the electron transport chain (ETC)), and diminished striatal neuroinflammation and apoptosis markers. Notably, the co-administration of trigonelline (TGN), an inhibitor of the nuclear factor erythroid-2-related factor 2 (Nrf2) pathway, significantly attenuated BBR’s protective effects, indicating that BBR’s neuroprotective actions are mediated via the Nrf2 pathway. These results underscore BBR’s potential in ameliorating motor impairments akin to PD, suggesting its promise in potentially delaying or managing PD symptoms. Further research is warranted to translate these preclinical findings into clinical settings, enhancing our comprehension of BBR’s therapeutic prospects in PD.

## 1. Introduction

Recent epidemiological research has linked environmental factors, including exposure to pesticides, with the onset of idiopathic Parkinson’s disease (PD) [1,2,3]. Rotenone (RTN), a hydrophobic compound derived from Leguminosae plant roots and utilized as a natural pesticide, easily penetrates the blood–brain barrier (BBB) and strongly inhibits mitochondrial complex I in the electron transport chain [4]. Studies have demonstrated a correlation between RTN exposure and increased PD risk in affected populations [1,2,3,4]. Moreover, RTN induces a spectrum of behavioral, neuropathological, and biochemical manifestations in rodents reminiscent of those observed in human PD, rendering it a valuable disease model for decades [4,5,6]. These manifestations include striatal nitrosative and oxidative stress, mitochondrial dysfunction, neuroinflammation, neurotransmitter deficiencies, significant neurodegeneration, and activation of the nuclear factor erythroid-2-related factor 2 (Nrf2) pathway [6,7,8,9]. In light of these findings, therapies employing antioxidants or anti-inflammatory agents have been proposed as potential mitigators of PD-like symptoms in animal models [7,8,9,10,11,12,13].

Berberine (BBR), a principal component in Chinese herbal remedies such as Rhizoma Coptidis, Rhizoma Cyperus, and Rhizoma Rhei, has traditionally served as an antimicrobial, antiprotozoal, and anti-infective agent against various fungal, yeast, parasite, and viral infections [14,15]. Recent evidence has increasingly highlighted BBR’s diverse pharmacological actions, encompassing anti-inflammatory, antifungal, antibacterial, antiviral, anti-tumor, anti-diabetic, anti-diarrheal, and anti-dyslipidemic effects [14,15,16,17]. Particularly, BBR is acknowledged as a potent antioxidant with the ability to traverse the BBB. It acts as a neuroprotective agent by scavenging reactive radicals, augmenting endogenous antioxidants, diminishing peroxidative reactions, and sustaining catalase activity within mitochondria via the Nrf2-mediated pathway. These mechanisms have shown efficacy against ischemia, stress, and toxin-induced behavioral impairments [8,18,19,20,21,22,23,24,25,26,27,28,29,30,31,32]. However, the impact of BBR on RTN-induced motor impairments remains unexplored. Therefore, this study aims to investigate the protective effects of BBR against RTN-induced motor impairments and neurotoxicity in rats, with a focus on the Nrf2-mediated pathway. Utilizing a well-established PD animal model, we evaluated motor impairments through assessments such as open-field, bar catalepsy, beam-crossing, rotarod, and grip strength measurements. Considering the documented association between striatal nitrosative and oxidative stress, mitochondrial dysfunction, neuroinflammation, and severe neurodegeneration with RTN-induced motor impairments [4,5,6,7,8,9,10,11,12,13], we also examined nitrosative and oxidative status, antioxidation potential, mitochondrial function, and markers of neuroinflammation and apoptosis in the striatum. Additionally, to elucidate the involvement of the Nrf2-mediated pathway in RTN treatment, trigonelline (TGN), an inhibitor of this pathway, was co-administered with BBR.

## 2. Materials and Methods

### 2.1. Animals

Animal handling and experimental procedures adhered strictly to the “Guidelines for the Care and Use of Laboratory Animals” established by the U.S. National Institutes of Health and were approved by the Institutional Animal Care and Use Committee of the National Taiwan University College of Medicine (IACUC Approval No: 20200519). Wistar rats, weighing 250–270 g and approximately 3 months old, were utilized in this study. These rats were group-housed, with three animals per Plexiglas cage, and provided ad libitum access to food and water. The housing environment was maintained at a controlled temperature of 22 ± 3 °C and operated on a 12/12-h light/dark cycle, with lights being activated at 7:00 am. All behavioral assessments were conducted during the animals’ active phase, specifically from 19:00 to 23:00 h. All experimental subjects were allowed to acclimatize to the laboratory environment with food and water available for 7 days before the experiment. The justification for the number of animals used was performed before the experiments and is included in the AICUC approval.

### 2.2. Drugs

Rotenone (RTN) (≥95%; Sigma, St. Louis, MO, USA), with the chemical name ((2R,6aS,12aS)-1,2,6,6a,12,12a-hexahydro-2-isopropenyl-8,9-dimethoxychromeno[3,4-b]furo[2,3-h]chromen-6-one), was prepared in a 1% DMSO solution and administered subcutaneously (s.c.). Berberine (BBR) (≥95%; Sigma, St. Louis, MO, USA), with the chemical name (9,10-Dimethoxy-7,8,13,13a-tetradehydro-2′H-[1,3]dioxolo[4′,5′:2,3]berbin-7-ium), was dissolved in double-distilled water and administered orally (p.o.). Trigonelline (TGN) (Sigma, St. Louis, MO, USA), with the chemical name (1-Methylpyridin-1-ium-3-carboxylate), was mixed in normal saline and administered intraperitoneally (i.p.). Freshly prepared drug solutions were used for each administration. Initial experiments with BBR at a low dose of 3 mg/kg did not yield noticeable effects; therefore, the dosage was incrementally increased to a maximum of 300 mg/kg to achieve statistically significant results. Dosages of 30 and 100 mg/kg were selected for this study based on their established effectiveness. All drug doses, derived from previous studies [8,9,18,25], were administered at a volume of 2 mL/kg body weight.

### 2.3. Experimental Groups and Drug Treatment

**Figure 1:**
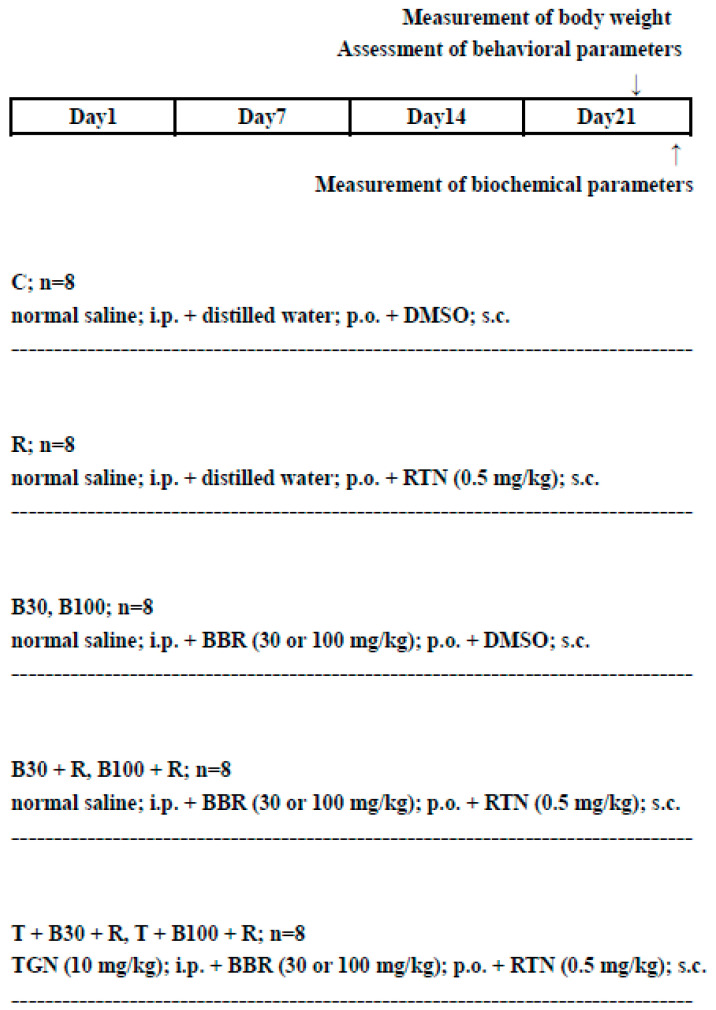
Experimental design and drug treatment paradigm.

The experimental rats were randomly divided into groups (n = 8 each, with a balanced representation of both sexes) as detailed in Table 1. Prior to injection of RTN or DMSO, administration of BBR or distilled water took place 60 min beforehand. TGN or normal saline injection preceded BBR administration by 30 min. On the 21st day, 8 h post-RTN or DMSO injection, all animals underwent behavioral assessment. The experimental layout is illustrated in Figure 1. To minimize subjective bias, each animal was assigned an arbitrary identification number. Behavioral assessments were conducted independently by two experienced coworkers who were unaware of the treatment regimen for each animal. Each experimenter conducted tests and evaluations separately. Animals were euthanized approximately 1 h after the behavioral assessments. During the study, nine rats succumbed to natural causes for reasons unknown.

### 2.4. Measurement of Body Weight

The body weight of the animals was measured both before RTN administration (1st day) and on the final day of the study (21st day). To calculate the percentage change in body weight, the following formula was applied: Change in body weight = (body weight on 1st day − body weight on 21st day)/body weight on 1st day × 100.

### 2.5. Open Field Test

To evaluate spontaneous locomotor activity, we conducted an open field test utilizing a wooden rectangular apparatus measuring 100 × 100 × 40 cm, with an activity detection device (Opto-Varimax-3 Units; Columbus Instruments, Columbus, OH, USA), colored light brown. The floor of the apparatus was partitioned into 25 rectangular squares demarcated by pencil lines. Illumination in the experimental room was provided by a 40 W white bulb situated 150 cm above the test apparatus. Each animal was placed in the center of the apparatus for a duration of 12 min, and the number of squares crossed during the final 10 min was recorded. A square crossing was tallied only when all four paws of the animal entered another square. Following each trial, the apparatus underwent thorough cleaning before the next animal was tested [33].

### 2.6. Bar Catalepsy Test

Cataleptic behavior was evaluated using the bar test, following the method outlined by Ferro et al. (2005) [8]. Rats were positioned in a standing posture with their forelimbs resting on a 10 cm high bar, and the time taken by each rat to remove one forelimb from the bar was recorded across three successive trials. A maximum time limit of 60 s was established for each trial. The total of the latencies observed in all three trials was computed to assess cataleptic behavior.

### 2.7. Beam-Crossing Task

To evaluate motor coordination, we employed a beam-crossing task wherein animals were required to traverse a narrow wooden beam. The beam comprised two platforms (8 cm in diameter) connected by a wooden beam measuring 0.5 mm in thickness, 2.0 cm in width, and 120 cm in length, elevated 50 cm above the ground. A box filled with sawdust was positioned beneath the beam to provide cushioning in case of falls. Rats were allotted 5 min for acclimation to the elevated beam prior to training. During a training trial, a rat was positioned at one end of the platform and encouraged to traverse the beam to reach the opposite end. The number of slips and the time taken to cross the beam in each trial were recorded [34].

### 2.8. Rotarod Activity

Motor coordination and grip performance were evaluated using a rotarod performance test. Before testing, rats underwent a training session to acquaint them with the task. During the test, rats were positioned on a rotating rod with a diameter of 7 cm, rotating at a speed of 25 rpm. The duration each rat remained on the rod was recorded, with a maximum time limit of 180 s [34].

### 2.9. Grip Strength Test

Neuromuscular strength was gauged through a grip strength test, following established protocols [35]. The apparatus comprised a 90 cm long metal wire with a diameter of 1 mm, horizontally affixed between two vertical supports and elevated 50 cm above a level surface. The animal was suspended by its forepaws at the midpoint of the wire and assessed according to the following criteria:

0—Fall off;
1—Hangs onto the wire using two forepaws;
2—Same as 1, but endeavors to climb on the wire;
3—Hangs onto the wire with two forepaws and one or both hind paws;
4—Hangs onto the wire with all forepaws plus the tail wrapped around the wire;
5—Escapes from the apparatus and falls onto the level surface.

### 2.10. Dissection and Homogenization

On the 21st day, around 1 h following behavioral assessment, rats were euthanized. The brain was promptly extracted, and the striatum was meticulously dissected, placed on ice, weighed, and homogenized using a 0.1 M phosphate buffer (pH 7.4). The homogenate was subsequently centrifuged at 10,000× *g* for 15 min, and portions of the resultant supernatant were collected and employed for biochemical analyses.

### 2.11. Estimation of Nitrite

To evaluate the accumulation of nitrite in the striatum supernatant, reflecting nitric oxide (NO) production, a colorimetric assay was conducted following the procedure outlined by Green et al. [35]. Equal volumes of the supernatant and Greiss reagent were mixed. The Greiss reagent consisted of 0.1% N-(1-naphthyl) ethylene diamine dihydrochloride, 1% sulfanilamide, and 2.5% phosphoric acid. The mixture was then incubated for 10 min at 25 °C in the dark. Subsequently, the absorbance was measured at 540 nm using a Shimadzu spectrophotometer (Kyoto, Japan). The concentration of nitrite in the supernatant was determined from a standard curve and expressed in μg/mL.

### 2.12. Assessment of Lipid Peroxidative Indices

The concentration of lipid peroxides was evaluated using the thiobarbituric acid reactive substances (TBARS) assay, adapted from Ohkawa et al. [36], and following the protocols delineated by Hashimoto et al. [37]. The concentration was quantified in nmol malondialdehyde per milligram of protein. Malondialdehyde levels were subsequently standardized to a standard preparation of 1,1,3,3-tetraethoxypropane.

### 2.13. Measurement of Glutathione (GSH)

GSH levels were determined using the method outlined by Ellman et al. [38]. To the homogenate, 10% trichloroacetic acid was added along with 1.0 mL of Ellman’s reagent, comprising 19.8 mg of 5,5′-dithiobis(2-nitrobenzoic acid) (DTNB) in 100 mL of 1.0% sodium citrate and 3 mL of phosphate buffer (pH 8.0). The mixture was subsequently centrifuged, and the absorbance of the resulting solution was measured at 412 nm. The outcomes were expressed in nmol GSH per milligram of tissue.

### 2.14. Measurement of Superoxide Dismutase (SOD) Activity

The assay to determine SOD activity relied on SOD’s capacity to impede the spontaneous oxidation of adrenaline to adrenochrome [39]. In this assay, 0.05 mL of the supernatant was mixed with 2.0 mL of carbonate buffer and 0.5 mL of EDTA. The reaction was initiated by adding 0.5 mL of epinephrine. The auto-oxidation of adrenaline (3 × 10^−4^ M) to adrenochrome at pH 10.2 was monitored by measuring the optical density at 480 nm. The alteration in optical density was recorded every minute and standardized against a blank reagent. SOD activity was expressed in units (milligrams per protein), with one unit of SOD activity causing approximately 50% inhibition of adrenaline. The outcomes were further expressed in nmol SOD units per milligram of tissue.

### 2.15. Measurement of Catalase (CAT) Activity

The CAT activity assay utilized in this study was adapted from the method outlined by Beers and Sizer [40]. In summary, a reaction mixture was prepared by combining 2 mL of phosphate buffer (pH 7.0), 0.95 mL of hydrogen peroxide (0.019 M), and 0.05 mL of supernatant, resulting in a total volume of 3 mL. The absorbance was then monitored at 240 nm every 10 s for 1 min. One unit of catalase (CAT) activity was defined as the quantity of enzyme required to decompose 1 mmol of peroxide per minute at 25 °C and pH 7.0. CAT activity was expressed in units per milligram of protein. Activity units were determined from a standard curve of H_2_O_2_. The findings were presented as catalase units per milligram of tissue.

### 2.16. Measurement of Mitochondrial Function

Mitochondria were isolated using a modified version of the differential centrifugation method as previously described [41]. Initially, a 10% homogenate of the striatum was prepared in ice-cold Tris-Sucrose buffer (0.25 M, pH 7.4) using a glass-Teflon grinder at 4 °C. The homogenate was then centrifuged at 1000× *g* for 10 min at 4 °C to obtain the nuclear pellet. The resulting supernatant underwent further centrifugation at 10,000× *g* for 20 min at 4 °C to isolate the mitochondrial pellet along with the cytosol. The pellet was washed three times in mannitol–sucrose–HEPES buffer (pH 7.4) and then resuspended in the same buffer. The activity of succinate dehydrogenase (SDH) was assessed following the method described by Pennington (1961) with slight modifications. In brief, mitochondrial protein (0.05 mg) was incubated with 50 mM potassium phosphate (pH 7.4) containing sodium succinate (0.01 mol/L) and p-iodonitrotetrazolium violet (2.5 µg/mL) for 10 min. The reaction was halted by adding 10% trichloroacetic acid (TCA). The resulting color was extracted using ethyl acetate/ethanol/trichloroacetic acid (5:5:1, *v*:*v*:*w*) and measured at 490 nm. SDH activity was expressed as the optical density (OD) value at 490 nm per milligram of protein. Total ATPase activity was determined by measuring the release of inorganic phosphate from ATP, following the method described by Prasad and Muralidhara (2013). The reaction was initiated by adding cytosolic protein (50 µg) to a reaction mixture containing Tris HCl buffer (0.02 M, pH 7.4), NaCl (100 mM), KCl (20 mM), and MgCl_2_ (5 mM), which was then incubated for 15 min at 37 °C. The reaction was stopped by adding 20% TCA. After centrifugation (15,009× *g*; 10 min), the phosphate content in the protein-free supernatant was estimated. Enzyme activity was expressed as micrograms of inorganic phosphate liberated per milligram of protein. NADH-cytochrome C reductase (complex I–III) and succinate-cytochrome C reductase (complex II–III) activities were determined following standard procedures [42].

### 2.17. Measurement of Neuroinflammatory Markers

The quantification of TNF-α, IL-1β, and IL-6 levels was carried out using an immunoassay kit (KRISHGEN BioSystem, Ashley Ct, Whittier, CA, USA). Specifically, a Quantikine rat TNF-α, IL-1β, and IL-6 immunoassay kit was employed. This kit utilizes a 4.5-h solid-phase sandwich enzyme-linked immunosorbent assay (ELISA) designed for the measurement of rat TNF-α, IL-1β, and IL-6 levels, employing a microplate reader. The concentrations of TNF-α, IL-1β, and IL-6 were determined from standard curves and expressed in picograms per milliliter of protein.

### 2.18. Caspase-3 Colorimetric Assay

Caspase-3, also known as CPP-32, Yama, or Apopain, is an intracellular cysteine protease. Typically, caspase-3 exists as an inactive pro-enzyme which then becomes activated during the apoptotic cascade. To evaluate the protease activity in tissue lysates/homogenates, a caspase-specific peptide linked to the color reporter molecule p-nitroaniline (pNA) is introduced. Caspase cleaves this peptide, liberating the chromophore pNA, which can be quantified spectrophotometrically at 405 nm. The colorimetric kit utilized for assessing caspase activity in this study was acquired from GeneTex Inc., Hsinchu, Taiwan. The findings were expressed as nanomoles of pNA per milligram of protein.

### 2.19. Determination of Protein

The protein content was assessed using the method of Lowry et al. (1951) [43].

### 2.20. Statistical Analysis

All results were reported as mean ± standard error of the mean (SEM). Statistical analyses were carried out using GraphPad Prism 8.3.0 (GraphPad Software Inc., San Diego, CA, USA). One-way ANOVA was employed followed by post hoc Tukey’s test for all behavioral and biochemical estimations. A *p*-value of less than 0.05 was deemed statistically significant, indicating notable differences between groups.

## 3. Results

Prior to RTN administration, the values of the assessed parameters exhibited considerable similarity across the various groups. No noteworthy differences were detected between the groups treated with BBR (30 or 100 mg/kg) and the control (C) groups for the examined parameters.

### 3.1. BBR Treatment Prevented RTN-Induced Decreases in Body Weight

**Figure 2:**
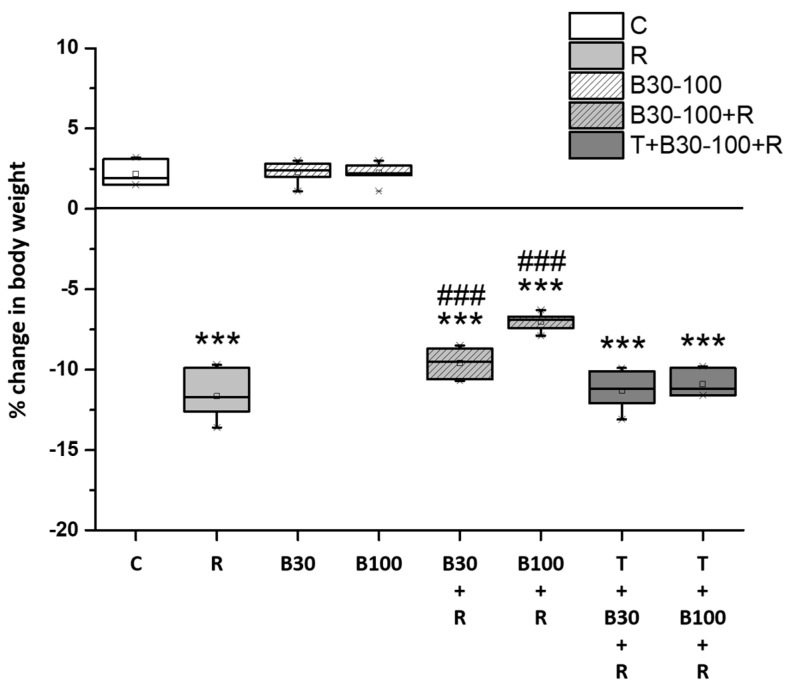
BBR prevented RTN-induced decreases in body weight in rats. Data are presented as mean ± SEM (n = 8). The measurements of the body weight were only assessed twice and the graph represents the total changes observed. One-way ANOVA with Tukey’s test: *** *p* < 0.001 as compared with C; ### *p* < 0.001 as compared with R. (C: control group; R: RTN 0.5 mg/kg treatment group; B30: 30 mg/kg BBR without RTN treatment group; B100: 100 mg/kg BBR without RTN treatment group; B30 + R: BBR 30 mg/kg + RTN treatment group; B100 + R: BBR 100 mg/kg + RTN treatment group; T + B30 + R: TGN 10 mg/kg + BBR 30 mg/kg + RTN treatment group; and T + B100 + R: TGN + BBR 100 mg/kg + RTN treatment group).

Treatment with RTN led to a notable decrease in body weight (−11.66% ± 1.43%, F = 667.31, *p* < 0.001). However, BBR treatment successfully mitigated the RTN-induced reduction in body weight, with a decrease of 2.06% in the B30 + R group (F = 99.14, *p* < 0.001) and 4.63% in the B100 + R group (F = 266.9, *p* < 0.001) (Figure 2). Additionally, TGN significantly abolished the protective effect of BBR against the RTN-induced reduction in the rats’ body weight.

### 3.2. BBR Treatment Prevented RTN-Induced Motor Impairment

**Figure 3:**
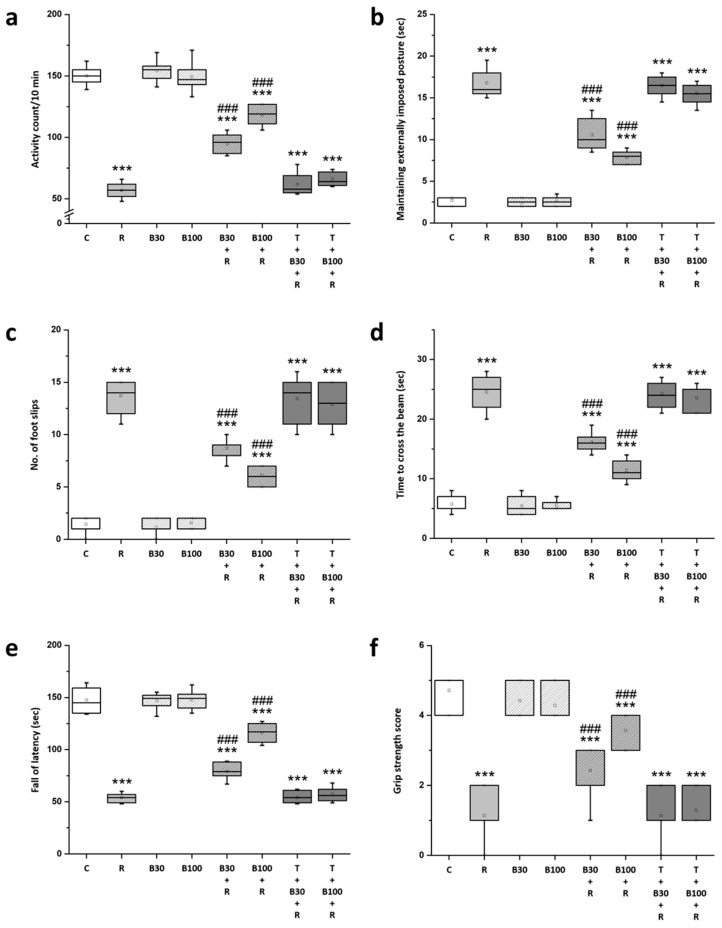
BBR prevented most RTN-induced motor impairment in the rats: (**a**) locomotor activity, (**b**) latency to remove from the bar (cataleptic behavior), (**c**) number of slips, (**d**) time taken to cross the beam (motor coordination), (**e**) latency to fall (motor coordination and grip performance), (**f**) grip strength score (neuromuscular strength). Data are presented as mean ± SEM (n = 8). One-way ANOVA with Tukey’s test: *** *p* < 0.001 as compared with C; ### *p* < 0.001 as compared with R. (C: control group; R: RTN 0.5 mg/kg treatment group; B30: 30 mg/kg BBR without RTN treatment group; B100: 100 mg/kg BBR without RTN treatment group; B30 + R: BBR 30 mg/kg + RTN treatment group; B100 + R: BBR 100 mg/kg + RTN treatment group; T + B30 + R: TGN 10 mg/kg + BBR 30 mg/kg + RTN treatment group; and T + B100 + R: TGN + BBR 100 mg/kg + RTN treatment group).

The current study also observed a decline in motor activity, catalepsy behavior, and coordination in animals following RTN treatment. In behavioral assessments in R groups, there was a decrease in: locomotor activity (from 150.14 ± 7.34 to 56.86 ± 6.12 count/10 min, F = 667.31, *p* < 0.001) (Figure 3a), latency to fall (from 147.57 ± 11.89 to 53.86 ± 4.22 s, F = 386.41, *p* < 0.001) (Figure 3e), and grip strength score (from 4.71 ± 0.49 to 1.14 ± 0.69, F = 125, *p* < 0.001) (Figure 3f); while there was an increase in: the latency to remove from the bar (from 2.71 ± 0.49 to 16.79 ± 1.6 s, F = 493.33, *p* < 0.001) (Figure 3b), the number of slips (from 1.43 ± 0.79 to 13.71 ± 1.6 slips, F = 331.16, *p* < 0.001) (Figure 3c), and the time taken to cross the beam (from 5.71 ± 1.38 to 24.57 ± 2.82 s, F = 252.52, *p* < 0.001) (Figure 3d).

BBR treatment significantly ameliorated the RTN-induced reduction in locomotor activity, latency to fall, and grip strength score, as well as the increase in the latency to remove from the bar, number of slips, and the time taken to cross the beam in both B30 + R groups (locomotor activity: from 56.86 ± 6.12 to 94.86 ± 8.03 count/10 min, F = 99.14, *p* < 0.001; latency to remove from bar: from 16.79 ± 1.6 to 10.57 ± 1.86 s, F = 61.22, *p* < 0.001; number of slips: from 13.71 ± 1.6 to 8.71 ± 0.95 slips, F = 50.34, *p* < 0.001; time taken to cross the beam: from 24.57 ± 2.82 to 16.14 ± 1.57 s, F = 47.68, *p* < 0.001; latency to fall: from 53.86 ± 4.22 to 79.43 ± 7.55 s, F = 61.22, *p* < 0.001; and grip strength score: from 1.14 ± 0.69 to 2.43 ± 0.79, F = 10.57, *p* < 0.001) and B100 + R groups (locomotor activity: from 56.86 ± 6.12 to 118.43 ± 7.87 count/10 min, F = 266.9, *p* < 0.001; latency to remove from bar: from 16.79 ± 1.6 to 7.86 ± 0.75 s, F = 178.23, *p* < 0.001; number of slips: from 13.71 ± 1.6 to 6.14 ± 0.9 slips, F = 118.69, *p* < 0.001; time taken to cross the beam: from 24.57 ± 2.82 to 11.43 ± 1.7 s, F = 110.88, *p* < 0.001; latency to fall: from 53.86 ± 4.22 to 116.29 ± 8.48 s, F = 304.09, *p* < 0.001; and grip strength score: from 1.14 ± 0.69 to 3.57 ± 0.53, F = 54.19, *p* < 0.001).

However, TGN significantly annulled the effect of BBR on RTN-induced changes in the behavior of the animals in the open-field test, bar catalepsy test, beam-crossing task, rotarod test, and grip strength test.

### 3.3. BBR Treatment Inhibited RTN-Induced Increases in Striatal Nitric Oxide and Lipid Peroxide Production

**Figure 4:**
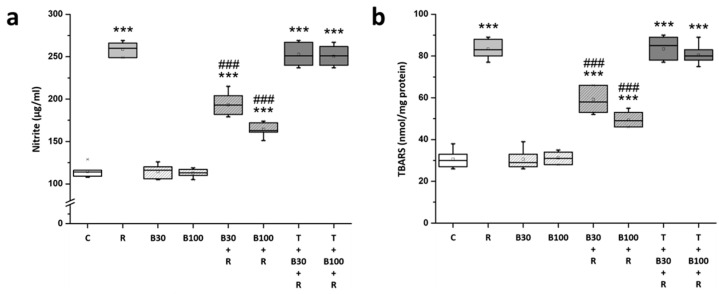
BBR reduced RTN-induced striatal nitrosative and oxidative stress in the rats: (**a**) nitrite and (**b**) TBARS. Data are presented as mean ± SEM (n = 8). One-way ANOVA with Tukey’s test: *** *p* < 0.001 as compared with C; ### *p* < 0.001 as compared with R. (C: control group; R: RTN 0.5 mg/kg treatment group; B30: 30 mg/kg BBR without RTN treatment group; B100: 100 mg/kg BBR without RTN treatment group; B30 + R: BBR 30 mg/kg + RTN treatment group; B100 + R: BBR 100 mg/kg + RTN treatment group; T + B30 + R: TGN 10 mg/kg + BBR 30 mg/kg + RTN treatment group; and T + B100 + R: TGN + BBR 100 mg/kg + RTN treatment group).

The levels of nitrite and TBARS were evaluated in rat striatal tissue. Post-hoc analysis revealed significant increases in both nitrite (by 125.81% from 114.57 ± 7.04 to 258.71 ± 7.72 μg/mL, F = 1331.99, *p* < 0.001) (Figure 4a) and TBARS (by 171.67% from 30.71 ± 4.07 to 83.43 ± 4.28 nmol/mg protein, F = 558.04, *p* < 0.001) (Figure 4b) levels in the rat striatum following RTN treatment.

The elevated nitrite and TBARS levels in R groups were significantly mitigated by BBR 30 mg/kg treatment (nitrite: by −45.49% to 193.14 ± 12.95 μg/mL, F = 132.37, *p* < 0.001; and TBARS: by −45.79% to 59.29 ± 6.26 nmol/mg protein, F = 70.93, *p* < 0.001) and by 100 mg/kg treatment (nitrite: by −65.21% to 164.71 ± 7.95 μg/mL, F = 503.64, *p* < 0.001; and TBARS: by −64.51% to 49.42 ± 3.51 nmol/mg protein, F = 264.69, *p* < 0.001). TGN nearly nullified the effect of BBR on RTN-induced increases in the striatal levels of nitric oxide and lipid peroxide production.

### 3.4. BBR Treatment Prevented RTN-Induced Decreases in Striatal Antioxidation Power

**Figure 5:**
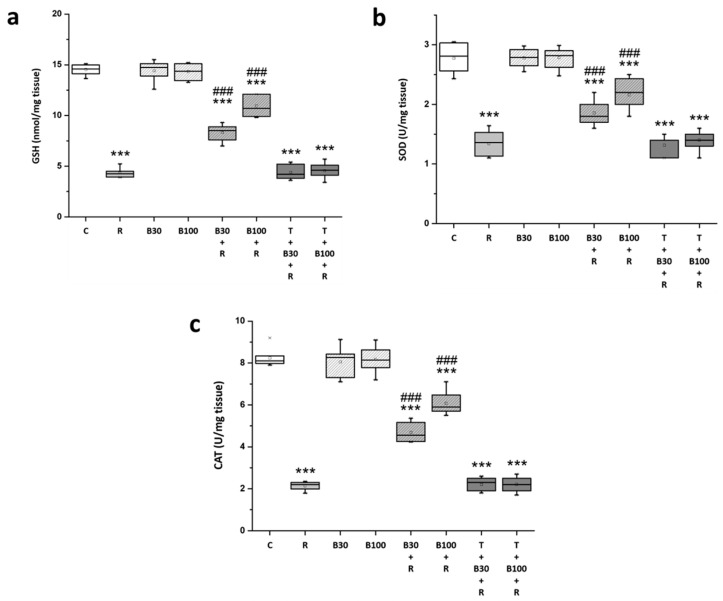
BBR prevented RTN-induced decreased striatal antioxidation power in the rats: (**a**) GSH, (**b**) SOD, and (**c**) CAT. Data are presented as mean ± SEM (n = 8). One-way ANOVA with Tukey’s test: *** *p* < 0.001 as compared with C; ### *p* < 0.001 as compared with R. (C: control group; R: RTN 0.5 mg/kg treatment group; B30: 30 mg/kg BBR without RTN treatment group; B100: 100 mg/kg BBR without RTN treatment group; B30 + R: BBR 30 mg/kg + RTN treatment group; B100 + R: BBR 100 mg/kg + RTN treatment group; T + B30 + R: TGN 10 mg/kg + BBR 30 mg/kg + RTN treatment group; and T + B100 + R: TGN + BBR 100 mg/kg + RTN treatment group).

After RTN treatment, the rats exhibited a significant decrease in the striatal levels of antioxidation power, including GSH (from 14.54 ± 0.52 to 4.32 ± 0.45 nmol/mg tissue, F = 1555.21, *p* < 0.001) (Figure 5a), SOD (from 2.77 ± 0.24 to 1.34 ± 0.2 U/mg tissue, F = 147.13, *p* < 0.001) (Figure 5b), and CAT (from 8.24 ± 0.6 to 2.13 ± 0.2 U/mg tissue, F = 1083.69, *p* < 0.001) (Figure 5c).

Meanwhile, the diminished GSH, SOD, and CAT levels in R groups were significantly restored by BBR 30 mg/kg treatment (GSH: by 39.33% to 8.34 ± 0.81 nmol/mg tissue, F = 131.47, *p* < 0.001; SOD: by 36.36% to 1.86 ± 0.2 U/mg tissue, F = 23.24, *p* < 0.001; and CAT: by 41.9% to 4.69 ± 0.44 U/mg tissue, F = 196.73, *p* < 0.001) and by 100 mg/kg treatment (GSH: by 64.97% to 10.96 ± 1 nmol/mg tissue, F = 255.94, *p* < 0.001; SOD: by 57.34% to 2.16 ± 0.25 U/mg tissue, F = 45.78, *p* < 0.001; and CAT: by 64.32% to 6.07 ± 0.56 U/mg tissue, F = 309.38, *p* < 0.001). TGN nullified the effect of BBR on RTN-induced decreases in the striatal levels of GSH, SOD, and CAT.

### 3.5. BBR Treatment Prevented RTN-Induced Striatal Mitochondrial Dysfunction

**Figure 6:**
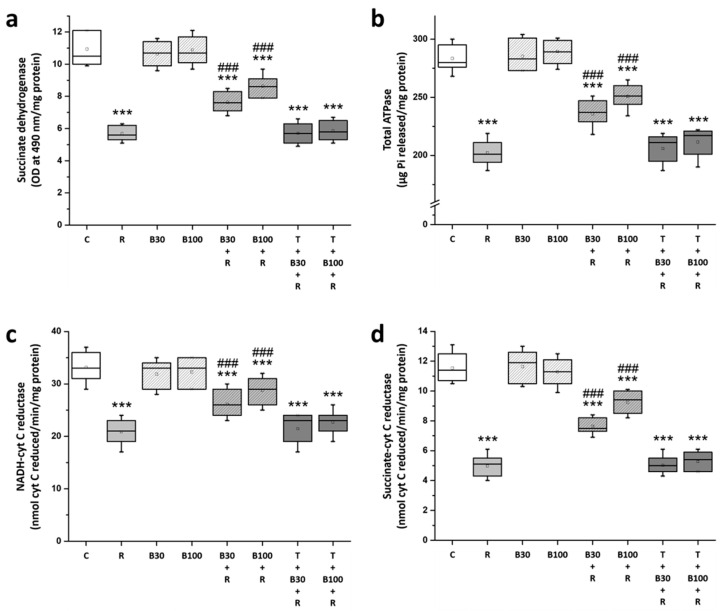
BBR prevented RTN-induced striatal mitochondrial dysfunction in the rats: (**a**) SDH, (**b**) total ATPase, (**c**) NADH-cytochrome C reductase (complex I-III), and (**d**) succinate-cytochrome C reductase (complex II-III). Data are presented as mean ± SEM (n = 8). One-way ANOVA with Tukey’s test: *** *p* < 0.001 as compared with C; ### *p* < 0.001 as compared with R. (C: control group; R: RTN 0.5 mg/kg treatment group; B30: 30 mg/kg BBR without RTN treatment group; B100: 100 mg/kg BBR without RTN treatment group; B30 + R: BBR 30 mg/kg + RTN treatment group; B100 + R: BBR 100 mg/kg + RTN treatment group; T + B30 + R: TGN 10 mg/kg + BBR 30 mg/kg + RTN treatment group; and T + B100 + R: TGN + BBR 100 mg/kg + RTN treatment group).

In Figure 6, a significant impairment of striatal mitochondrial function in R rats is highlighted, indicated by a substantial decrease in the levels of SDH (from 10.94 ± 1.04 to 5.69 ± 0.46 OD at 490 nm/mg protein, F = 149.86, *p* < 0.001) (Figure 6a), total ATPase (from 283.43 ± 11.1 to 202.43 ± 11.34 μg Pi released/mg protein, F = 182.31, *p* < 0.001) (Figure 6b), NADH-cytochrome C reductase (from 33.14 ± 2.73 to 20.86 ± 2.41 nmol cyt C reduced/min/mg protein, F = 79.53, *p* < 0.001) (Figure 6c), and succinate-cytochrome C reductase (from 11.54 ± 0.94 to 4.97 ± 0.73 nmol cyt C reduced/min/mg protein, F = 213.09, *p* < 0.001) (Figure 6d) as compared to the C group by Post-hoc analysis.

BBR significantly alleviated RTN-induced striatal mitochondrial dysfunction; the diminished levels of SDH, total ATPase, NADH-cytochrome C reductase, and succinate-cytochrome C reductase in R groups were significantly elevated by BBR 30 mg/kg treatment (SDH: by 36.95% to 7.63 ± 0.61 OD at 490 nm/mg protein, F = 45.26, *p* < 0.001; total ATPase: by 41.09% to 235.71 ± 11.15 μg Pi released/mg protein, F = 30.67, *p* < 0.001; NADH-cytochrome C reductase: by 43% to 26.14 ± 2.67 nmol cyt C reduced/min/mg protein, F = 15.1, *p* < 0.001; and succinate-cytochrome C reductase: by 40.64% to 7.64 ± 0.54 nmol cyt C reduced/min/mg protein, F = 61.03, *p* < 0.001) and by 100 mg/kg treatment (SDH: by 56.19% to 8.64 ± 0.65 OD at 490 nm/mg protein, F = 96, *p* < 0.001; total ATPase: by 59.6% to 250.71 ± 10.16 μg Pi released/mg protein, F = 70.39, *p* < 0.001; NADH-cytochrome C reductase: by 63.93% to 28.71 ± 2.56 nmol cyt C reduced/min/mg protein, F = 34.9, *p* < 0.001; and succinate-cytochrome C reductase: by 64.84% to 7.64 ± 0.54 nmol cyt C reduced/min/mg protein, F = 120.66, *p* < 0.001). However, TGN significantly blocked the effect of BBR on RTN-induced decreases in the striatal levels of SDH, total ATPase, NADH-cytochrome C reductase, and succinate-cytochrome C reductase.

### 3.6. BBR Treatment Inhibited RTN-Induced Increases in Striatal Neuroinflammatory and Apoptotic Markers

**Figure 7:**
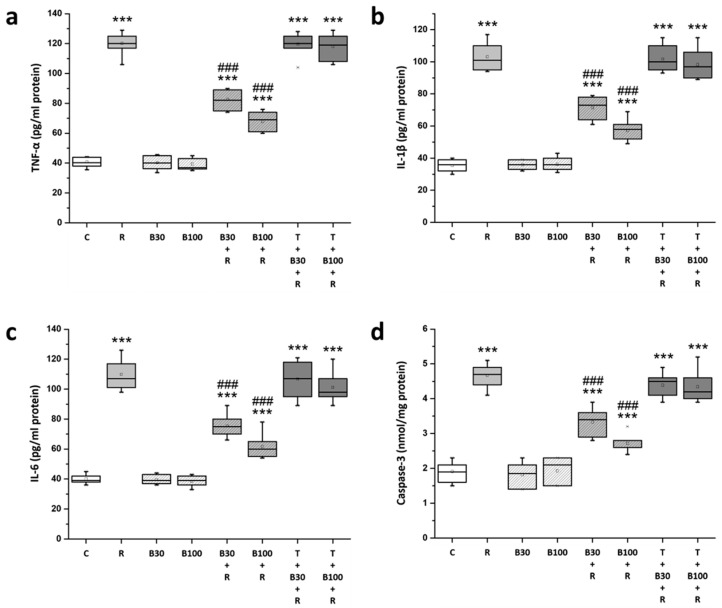
BBR blocked RTN-induced increased striatal neuroinflammatory and apoptotic markers in the rats: (**a**) TNF-α, (**b**) IL-1β, (**c**) IL-6 and (**d**) caspase-3. Data are presented as mean ± SEM (n = 8). One-way ANOVA with Tukey’s test: *** *p* < 0.001 as compared with C; ### *p* < 0.001 as compared with R. (C: control group; R: RTN 0.5 mg/kg treatment group; B30: 30 mg/kg BBR without RTN treatment group; B100: 100 mg/kg BBR without RTN treatment group; B30 + R: BBR 30 mg/kg + RTN treatment group; B100 + R: BBR 100 mg/kg + RTN treatment group; T + B30 + R: TGN 10 mg/kg + BBR 30 mg/kg + RTN treatment group; and T + B100 + R: TGN + BBR 100 mg/kg + RTN treatment group).

Compared with the C groups, striatal levels of TNF-α (from 40.19 ± 3.13 to 120.14 ± 9.14 pg/mL protein, F = 213.09, *p* < 0.001) (Figure 7a), IL-1β (from 35.43 ± 3.64 to 103.14 ± 8.71 pg/mL protein, F = 656.99, *p* < 0.001) (Figure 7b), IL-6 (from 40.14 ± 3.02 to 109.86 ± 9.91 pg/mL protein, F = 360.25, *p* < 0.001) (Figure 7c), and caspase-3 (from 1.91 ± 0.29 to 4.67 ± 0.33 nmol/mg protein, F = 279.37, *p* < 0.001) (Figure 7d) were significantly increased in R groups; these increased TNF-α, IL-1β, IL-6, and caspase-3 levels in R groups were significantly inhibited by BBR 30 mg/kg treatment (TNF-α: by 46.82% to 82.71 ± 7.3 pg/mL protein, F = 98.72, *p* < 0.001; IL-1β: by 47.05% to 71.29 ± 6.9 pg/mL protein, F = 57.58, *p* < 0.001; IL-6: by 49.4% to 75.43 ± 7.59 pg/mL protein, F = 53.27, *p* < 0.001; and caspase-3: by 48.91% to 3.33 ± 0.39 nmol/mg protein, F = 48.28, *p* < 0.001) and by 100 mg/kg treatment (TNF-α: by 66.10% to 67.29 ± 6.6 pg/mL protein, F = 199.44, *p* < 0.001; IL-1β: by 67.72% to 57.29 ± 6.7 pg/mL protein, F = 121.94, *p* < 0.001; IL-6: by 69.26% to 61.57 ± 8.14 pg/mL protein, F = 99.26, *p* < 0.001; and caspase-3: by 71.01% to 2.71 ± 0.25 nmol/mg protein, F = 154.27, *p* < 0.001). TGN significantly abolished the protective effect of BBR on RTN-induced increases in the striatal levels of TNF-α, IL-1β, IL-6, and caspase-3.

## 4. Discussion

The study underscores the protective role of BBR against RTN-induced motor deficits, nitrosative and oxidative stress, mitochondrial dysfunction, neuroinflammation, and apoptotic activation, possibly through the Nrf2-mediated pathway. These findings suggest that BBR may hold promise for the treatment of Parkinson’s disease (PD) in humans. RTN treatment has long been established as a reliable method for inducing neurotoxicity in animal models of PD, offering experimental advantages over other models [5,6,9]. Unlike acute toxin models, RTN administration more closely mimics the chronic progression of PD observed in patients, providing insight into the selective vulnerability of nigrostriatal degeneration. This model enables the investigation of various molecular and biochemical processes underlying PD pathology and clinical manifestations [6,9].

In this study, RTN administration resulted in significant reductions in body weight, locomotor activity, rotarod performance, grip strength, and motor coordination in rats, indicative of neurotoxicity and clinical PD symptoms. These findings are consistent with previous research demonstrating RTN-induced impairments in behavioral parameters, oxidative defense, mitochondrial function, and neurotransmitter levels due to dopaminergic degeneration [4,5,6,7,8,9,10,11,12,13]. BBR treatment mitigated most of the RTN-induced motor impairments, implicating the Nrf2-mediated pathway in its neuroprotective effects [5,6]. However, co-administration of TGN significantly attenuated the protective effects of BBR on behavioral outcomes. Although this study did not assess DA neuron damage in the substantia nigra pars compacta (SNpc) or striatal histopathology in RTN-treated rats, previous studies have linked RTN-induced motor deficits to altered DA neuronal activity in the SNpc and striatum resulting from neuronal injury or death [6,7,8,9,10,11,12,13].

Previous studies have consistently demonstrated a strong correlation between RTN-induced motor impairment and alterations in striatal nitrosative and oxidative status [6,7,8,9,10,11,12,13]. Similarly, our present study revealed that RTN administration led to increased levels of nitrite and TBARS, along with decreased levels of GSH, SOD, and CAT in the rat striatum. Moreover, RTN adversely affected the levels of SDH, ATPase, and ETC enzymes, indicating multiple functional impairments in mitochondrial function [4,7,8,9,44]. These findings further support the involvement of nitrosative and oxidative stress, as well as mitochondrial dysfunction, in RTN-induced motor impairment. As a specific inhibitor of complex I in the electron transport chain, RTN disrupts mitochondrial respiration and enhances ROS production [4,7,8,9]. Additionally, mitochondrial dysfunction depletes ATP, depresses Na+/K(+)-ATPase activity, and causes graded neuronal depolarization; it also relieves the voltage-dependent Mg2+ block of the NMDA receptor, which is highly permeable to Ca^2+^, then triggers NMDA receptor activation, exacerbating mitochondrial impairment [45,46]. NMDA receptor hyperactivity leads to calcium influx and ROS/RNS formation, resulting in lipid peroxidation and DNA damage [47,48]. The reciprocal interaction between mitochondria and NMDA receptors exacerbates nitrosative and oxidative stress induced by RTN. Furthermore, NO inhibits key enzymes of energy metabolism and exacerbates ROS/RNS production, causing further cellular damage [7,9,13,49]. These findings underscore the involvement of both nitrosative and oxidative stress and mitochondrial dysfunction in RTN-induced behavioral impairment.

Excessive oxidative stress triggers an inflammatory response and prompts the release of inflammatory mediators such as TNF-α, IL-1β, and IL-6, initiating the apoptotic pathway. This inflammatory cascade is considered a key mechanism underlying neuronal cell death and PD progression [7,22]. Consistent with previous reports, our study observed elevated levels of TNF-α, IL-1β, and IL-6 in the striatum of RTN-treated rats, indicating the involvement of neuroinflammatory processes in RTN-induced neurotoxicity and motor dysfunction [7,9,10,12,13]. Moreover, the increased levels of caspase-3 after RTN treatment suggest the activation of the apoptotic cascade in the rat striatum, consistent with previous findings in RTN-induced PD animal models exhibiting increased apoptosis and reduced neural cell numbers [7,10,11,12,13]. In the present study, BBR significantly attenuated RTN-induced mitochondrial dysfunction, nitrosative and oxidative damage, neuroinflammation, and apoptosis in the striatum. Co-administration of TGN nearly abolished the protective effects of BBR on biochemical parameters, suggesting that BBR may exert neuroprotection in the striatum by interacting with the Nrf2-mediated pathway to counteract RTN-induced behavioral impairments.

Nrf2, a transcription factor known for inducing the expression of antioxidative, anti-inflammatory, and pro-survival genes, plays a crucial role in protecting against oxidative and inflammatory responses in experimental models of PD [22,50,51]. It promotes the upregulation of antioxidative proteins while also binding to inflammatory cytokine genes to inhibit their transcription, thus eliminating ROS and exerting anti-inflammatory and anti-apoptotic effects. Moreover, the Nrf2-mediated pathway has been extensively documented to be involved in maintaining mitochondrial function in in vitro models of PD [8,50,51,52,53,54]. Consistent with previous findings, our experimental results demonstrate that the protective effects of BBR are mediated through the Nrf2-mediated pathway, and suppression of this pathway abolishes the protective effects of BBR [8,19,20,28,30]. These protective effects are often associated with the induction of heme oxygenase-1 (HO-1) through the Nrf2-mediated pathway [19,20,28]. Additionally, upstream mediators such as phosphatidylinositol 3-kinase (PI3K)/Akt and p38 are involved in activating the Nrf2-mediated pathway. Studies have highlighted the role of the PI3K/Akt pathway in inducing the Nrf2-mediated pathway and subsequent neuroprotective effects of BBR [19,23,24]. It appears that BBR’s antioxidant potential primarily relies on its modulation of the Nrf2-mediated pathway via the activation of PI3K/Akt, p38, and HO-1 expressions, thereby enhancing the antioxidant defense system, protecting against oxidative damage, and reducing apoptosis [8,19,20,28,30]. However, further studies are warranted to fully understand the modulatory impact of BBR on the Nrf2-mediated pathway.

In our study, BBR effectively reduced the elevated levels of nitrite, TBARS, TNF-α, IL-1β, IL-6, and caspase-3, while concurrently upregulating the levels of SDH, ATPase, ETC enzymes, GSH, SOD, and CAT in the rat striatum following RTN treatment. This multifaceted action of BBR suggests its involvement in various pathophysiological pathways. However, the protective effects of BBR were nullified by TGN, indicating that BBR may confer neuroprotection against RTN-induced neurotoxicity, possibly through the Nrf2-mediated pathway. These findings may shed light on the mechanisms underlying BBR’s ability to prevent RTN-induced motor deficits, as evidenced by our behavioral assessments, although further elucidation is warranted.

## 5. Conclusions

In conclusion, our study highlights the therapeutic potential of BBR in treating PD using an animal model. We propose that BBR exerts its neuroprotective effects through anti-oxidative, mitochondrial dysfunction prevention, anti-neuroinflammatory, and anti-apoptotic pathways, possibly mediated by the Nrf2 signaling pathway. These findings emphasize the need for further research to translate these preclinical findings into clinical studies, aiming to better understand the exact mechanism of action of BBR. Such investigations hold promise for the development of novel treatments for human PD. Future preclinical and clinical studies should explore the role of BBR in treating clinically relevant human PD and elucidate its potential benefits in clinical settings, including immunostaining showing the nuclear localization of Nrf2 in the brain, immunohistochemical analyses of the substantia nigra and striatum, which could provide direct evidence of BBR’s neuroprotective effects on dopaminergic neurons, and potential synergistic effects of BBR with other established PD treatments, such as levodopa or dopamine agonists etc.

## Figures and Tables

**Table 1:** Experimental groups.

Experimental		Treatment	n = 8
Group			♂:♀ = 4:4
1.	**C**	control (normal saline; i.p. + distilled water; p.o. + DMSO; s.c.) for 21 days	
2.	**R**	normal saline; i.p. + distilled water; p.o. + RTN (0.5 mg/kg; s.c.) for 21 days	
3.	**B30**	normal saline; i.p. + BBR (30 mg/kg; p.o.) + DMSO; s.c. for 21 days	
4.	**B100**	normal saline; i.p. + BBR (100 mg/kg; p.o.) + DMSO; s.c. for 21 days	
5.	**B30+R**	normal saline; i.p. + BBR (30 mg/kg; p.o.) + RTN (0.5 mg/kg; s.c.) for 21 days	
6.	**B100+R**	normal saline; i.p. + BBR (100 mg/kg; p.o.) + RTN (0.5 mg/kg; s.c.) for 21 days	
7.	**T+B30+R**	TGN (10 mg/kg; i.p.) + BBR (30 mg/kg; p.o.) + RTN (0.5 mg/kg; s.c.) for 21 days	
8.	**T+B100+R**	TGN (10 mg/kg; i.p.) + BBR (100 mg/kg; p.o.) + RTN (0.5 mg/kg; s.c.) for 21 days	

Note: BBR: berberine; i.p.: intraperitoneally; p.o.: orally; RTN: rotenone; s.c.: subcutaneously; TGN: trigonelline.

## Data Availability

The data presented in this study are available on request from the corresponding author. The data are not publicly available due to privacy considerations.
